# Meteorological Table

**Published:** 1813-08

**Authors:** 


### METEOROLOGICAL TABLE.

### *From June 25, to July 25,* 1813.

| | D. | Therm. | | | Barom. | | Hygrom. *Dry.* | | | *Damp.* | | | Winds. | Atmos. Variation. |
|---|---|---|---|---|---|---|---|---|---|---|---|---|---|---|
| | 26 | 63 | 73 | 62 | $30^2$ | — | 20 | 38 | 15 | | | | E. | F.... |
| | 27 | 65 | 76 | 62 | $30^2$ | $^1$ | 28 | 43 | 24 | | | | E. | F.... |
| | 28 | 63 | 68 | 60 | 30 | $29^9$ | 15 | 21 | 7 | | | | N.SE. | F.. C.. R.. |
| ● | 29 | 64 | 67 | 57 | 30 | $29^8$ | 17 | 15 | 10 | | | | S.SW... | F..R..F..—..R...inN |
| | 30 | 51 | 54 | 57 | | $29^7$ | 3 | | | | 11 | 5 | SW.. | R.. — ... — ... |
| | 1 | 53 | 62 | 57 | $29^8$ | — | | | | 9 | 7 | 1 | W.. | R..—...—...R... in N |
| | 2 | 54 | 65 | 53 | $29^9$ | — | 4 | 15 | 5 | | | | NW.N.. | F.. — ... C.. R. |
| | 3 | 54 | 65 | 53 | 30 | '$30^1$ | 5 | — | 6 | | | | N. | F.. C... |
| | 4 | 58 | 67 | 57 | $30^2$ | — | 10 | 21 | 12 | | | | N. | F... |
| ☽ | 5 | 56 | 68 | 57 | | | 15 | 26 | 15 | | | | N.S.. | F.. |
| | 6 | 62 | 70 | 59 | $30^1$ | $29^9$ | 15 | 38 | 27 | | | | SW.. | F... F.... |
| | 7 | 69 | 79 | 55 | $29^9$ | $^8$ | 21 | 40 | 27 | | | | SW.. | F.... C.. |
| | 8 | 65 | 66 | 61 | $29^8$ | $^7$ | 24 | 20 | 17 | | | | SW. | F..' |
| | 9 | 66 | 76 | 63 | $29^8$ | — | 15 | 37 | 26 | | | | W. | F... |
| | 10 | 65 | 75 | 63 | $29^9$ | 30 | 24 | 41 | 27 | | | | W.. | F... |
| | 11 | 62 | 69 | 62 | | | 20 | 25 | 21 | | | | W. | F.. |
| | 12 | 66 | 77 | 63 | $29^9$ | — | 28 | 41 | 26 | | | | S.W.N. | F... |
| ○ | 13 | 65 | 71 | 61 | $29^9$ | $^8$ | 20 | 21 | 15 | | | | NE.S. | F.. R.. in N |
| | 14 | 64 | 70 | 62 | $29^8$ | $^7$ | 10 | 6 | 2 | | | | SW. | F... C.. R —. in N |
| | 15 | 60 | 55 | 57 | | | 5 | | | | 10 | 1 | W. | F. R...R...R.. |
| | 16 | 62 | 73 | 59 | $29^9$ | — | 9 | 11 | 16 | | | | W. | F.. R.. F.. |
| | 17 | 61 | 71 | 60 | $29^9$ | 30 | 11 | 24 | 16 | | | | W. | F.. R. F... |
| | 18 | 61 | 71 | 60 | 30 | $29^9$ | 25 | — | 26 | | | | SW. | F... |
| | 19 | 63 | 66 | 60 | | $29^7$ | 14 | 15 | — | | | | SW.N.SE. | C. R.. C.. |
| ☾ | 20 | 65 | 75 | 64 | $29^6$ | — | 10 | 42 | 20 | | | | W.. | F.... R. |
| | 21 | 64 | 70 | 63 | $29^7$ | — | 12 | 17 | 9 | | | | SW. | F.. R. |
| | 22 | 62 | 71 | 61 | $29^6$ | — | 14 | 20 | 10 | | | | N E. W. | F... R. |
| | 23 | 63 | 70 | 59 | $29^6$ | — | 11 | 26 | 14 | | | | N W.S.. | F... R... F. |
| | 24 | 57 | 72 | 66 | | $29^7$ | 8 | 30 | — | | | | S.SW. | C..R...—...—...inN |
| | 25 | 60 | 70 | 66 | $29^5$ | -$29^6$ | 30 | 15 | 10 | | | | W... | F.. R.. F.. R... F... |

Quantity of rain from the 25th of June to the 25th of July, 2 inches $\frac{45}{100}$.

28th. Cold fog in the night; distant thunder and flashes of lightning in the evening, with some rain. Temperature sensibly more warm in the evening than in the morning, though the thermometer indicates three degrees less of heat.—29th. Gale from the SW in the night, with rain; distant thunder in the evening.

2d. Stormy wind from N in the morning, with cold rain.—7th. Heat oppressive.—15th. Rain the whole day. St. Swithin.—19th. Variable winds, light and soft, passing round the compass, with showers in the middle of the day.—23d. Thunder in the afternoon, with heavy showers.—24th. Thunder, with heavy showers, P.M. Much rain in the night.

Inflammatory affections of the throat and chest have still occasionally appeared; and in one instance of pneumonic inflammation, the cause was evidently traced to the application of highly heated air to the lungs. A few cases of cholera have occurred.

*Prince's Street, Cavendish Square.*

MONTHLY

